# Chiral-at-Tungsten Dioxo Complexes—A Computational Study on Inhibiting Racemization

**DOI:** 10.1021/acs.inorgchem.5c01280

**Published:** 2025-05-29

**Authors:** George Dhimba, Alfred Muller, Koop Lammertsma

**Affiliations:** † Department of Chemical Sciences, 61799 University of Johannesburg, Auckland Park, Johannesburg 2006, South Africa; ‡ Department of Chemistry and Pharmaceutical Sciences, Faculty of Sciences, 1190 Vrije Universiteit Amsterdam, De Boelelaan 1108, 1081 HZ Amsterdam, The Netherlands

## Abstract

Chiral cis-WO_2_(acac)_2_ and cis-WO_2_(nacnac)_2_ complexes racemize via four pathways according to DFT calculations at ωB97X-D/6–311+G­(2d,f) with LANL2DZ for W and with inclusion of acetonitrile solvent. Steric congestion by N-Me and N-Ph substitution of the two nacnac ligands has a substantial geometrical impact and raises the barriers for all pathways. Despite this, even all N-Me and all N-Ph substituted derivatives of cis-WO_2_(nacnac)_2_ have the same four channels for racemization. For each of these complexes, the Dhimba-Muller-Lammertsma (DML) twist is preferred over the Conte-Hippler (CH) twist and significantly favored over the established Bailar (B) and Ray-Dutt (RD) twists. The favored DML barrier for WO_2_(nacnac)_2_^Ph4^ has a large estimated Δ*G* barrier of 25.7 kcal/mol, suggesting it to be a viable chiral-at-tungsten complex for asymmetric catalysis.

## Graphical abstract

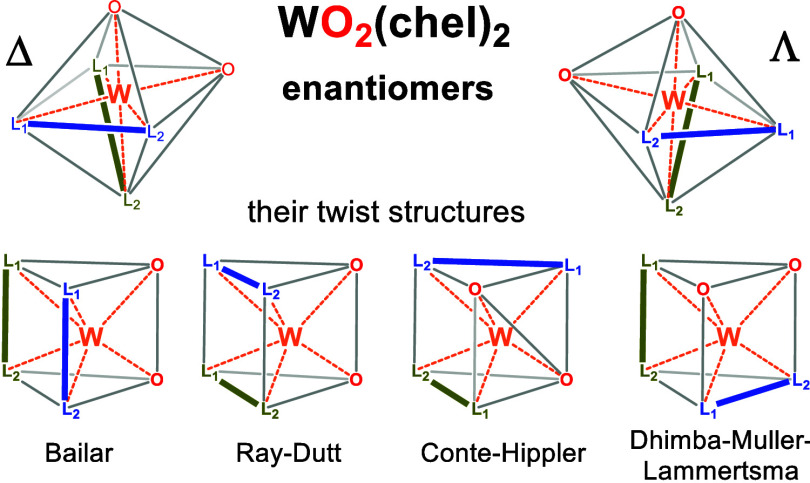

## Introduction

Chelated transition metal complexes used in asymmetric catalysis typically have chiral ligands.^1–15^ At first sight, this may seem counterintuitive as these are already Λ or Δ chiral-at-metal, making them diastereomeric, but the common perception is that they readily racemize at the metal center.^16^ Recent years, however, have witnessed the emergence of stable achiral ligand catalysts that are exclusively chiral-at-metal.^17–26^ These catalysts have demonstrated versatility in a range of organic transformations, providing superior chiral induction due to the proximity between the chiral metal center and the substrate during catalytic reactions.^24–32^

So far, chiral-at-metal catalysis has been dominated by noble metal complexes, such as those of Ru, Ir, Rh, and Os, with relatively few reports on other metals, presumably due to their tendency to racemize in solution.^26,28–31,33–35^ Broadening the scope of catalytic applications to include other transition metals commonly used in homogeneous catalysis would be highly beneficial. Achieving this goal requires a deeper understanding of the racemization process and strategies to inhibit it.

Despite decades of research, much remains to be learned. Over half a century ago, Muetterties reported detailed topological analyses on the racemization of 5- and 6-coordinated complexes,^36–38^ but the follow-up studies have been limited. Until recently, it was widely believed that racemization of octahedral complexes occurred exclusively via the Bailar (B) and Ray-Dutt (RD) twist mechanisms (Figure 1a,b).^39,40^ However, in 2016, Conte and Hippler (CH) reported an alternative twist for cis-MoO_2_(acac)_2_ (acac = acetylacetonate) (Figure 1c).^41^ More recently, we reported yet another twist (DML) with an even lower racemization barrier for this complex (Figure 1d).^42^ Importantly, the DML barrier aligns best with the value determined experimentally by NMR spectroscopy.^41^ Still, the universality of the CH and DML twists remains to be established. While we have demonstrated their prevalence in MoO_2_(nacnac)_2_ (nacnac = β-diketiminate)^43^ and MoO_2_(acnac)_2_ (acnac = β-ketoiminate),^44^ other metals have yet to be explored. This study aims to investigate tungsten complexes.

**Figure 1:**
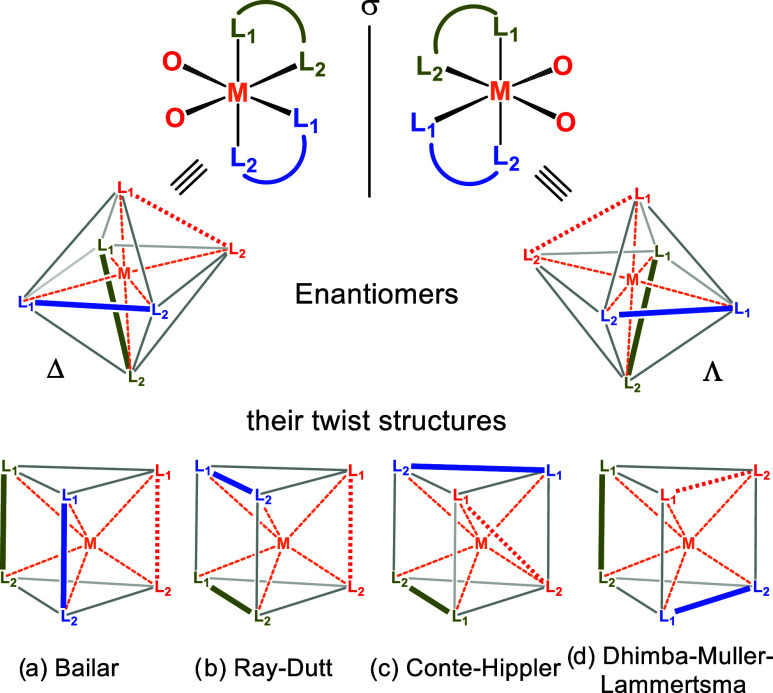
(a) Bailar, (b) Ray-Dutt, (c) Conte-Hippler, and (d) Dhimba-Muller-Lammertsma twists by which chiral octahedral dioxo complexes undergo racemization. The bidentate ligands are shown in blue and green, and the two oxo ligands with a dashed line, colored red. The gray lines complement the edges of the octahedral and trigonal prismatic structures, with the dashed orange lines representing the transition metal coordination sites.

It is instructive to recall Muetterties’ topological graph for MX_2_(chel)_2_, as shown in Figure 2 (left).^36^ This graph illustrates the various stereochemical pathways for racemization, with connecting paths labeled according to the associated twist mechanisms. Our computational survey of MoO_2_(acac)_2_ and MoO_2_(nacnac)_2_ showed full compliance with Muetterties’ graph, confirming its utility as a predictive tool for racemization and revealing the DML twist to represent the lowest energy barrier by far for racemization.^42,43^ The CH twist, which is the next best, can be right-handed (Δ) and left-handed (Λ) with the transition structure representing a valley-ridge inflection point connecting to the high-energy trans isomer.

**Figure 2:**
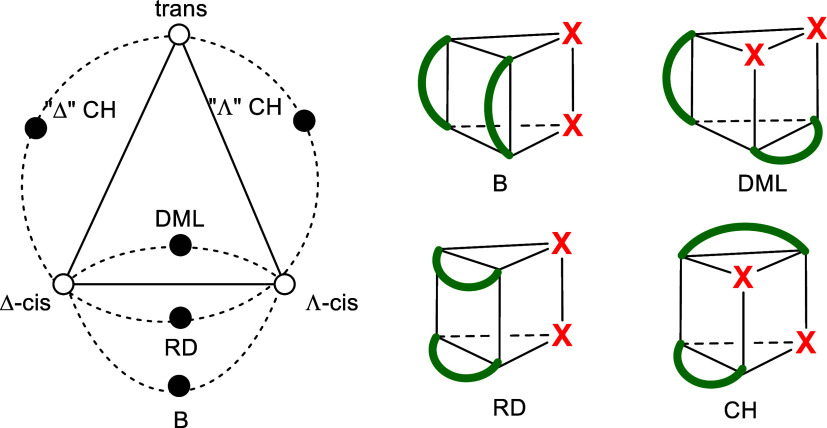
Muetterties’ adapted topological representation of MX_2_(chel)_2_ with octahedral structures (open dots) and connecting trigonal prismatic ones (closed dots) shown separately with green curved lines for the chelates and labeled with the twist mechanism, B= Bailar, RD = Ray-Dutt, CH = Conte-Hippler, and DML = Dhimba-Muller-Lammertsma.

Tungsten, like its lighter congener molybdenum, is used in catalytic applications like oxidation,^45–48^ alkene and alkyne metathesis,^49–53^ hydrogenation,^54–58^ and CO_2_ coupling reactions.^59–62^ Tungsten’s heavier atomic weight and deeper d-orbitals make its complexes more stable in higher oxidation states, such as WO_2_(acac)_2_ and WO_2_(acnac)_2_. These complexes are less prone to reduction^17^ and dimerization,^63^ and their crystal structures have been reported.^64–66^ Building on our earlier studies on MoO_2_(chel)_2_ (chel = chelate) complexes, this work seeks to achieve three objectives: (a) to establish the merits of CH and DML twists for WO_2_(chel)_2_ complexes, (b) to determine the relative energy barriers of all the twist mechanisms, and (c) to assess the feasibility of developing chiral-at-tungsten catalysts.

## Computational Details

The potential energy surface for all minima and transition structures was examined with Gaussian 16 version B01 ^67^ using the hybrid meta-GGA functional ωB97X-D,^68^ which incorporates empirical dispersion terms and long-range interactions.^69^ The 6–31G­(d) basis set was used for C, H, O, and N, and the LANL2DZ pseudopotential for W.^70–72^ Frequency calculations were used to verify the nature of all stationary points with zero and one imaginary frequency for minima and transition structures, respectively. The energies obtained for single-point calculations with the larger 6–311+G­(2d,p) basis are used throughout. For the WO_2_(nacnac)_2_ systems, the effects of solvation (acetonitrile) were included using the PCM model.^73–75^

## Results and Discussion

The paper is structured as follows. We begin by discussing the racemization of WO_2_(acac)_2_ and compare it to that of MoO_2_(acac)_2_. Next, we analyze the twisting motions of WO_2_(nacnac)_2_ and compare them to the molybdenum analogue. Following this and in search of a viable nonracemizing chiral-at-tungsten complex, we consider the effect of N-substitution of the nacnac ligands by methyl and phenyl groups to influence the magnitude of the racemization barriers.

### cis-WO_2_(acac)_2_

The global cis-WO_2_(acac)_2_ minimum has a distorted octahedral geometry (Δin Figure 3) like that of MoO_2_(acac)_2_, but its structure is a bit tighter. For instance, both W–O bond lengths of 2.005 Å (trans-O (O_t_)) and 2.224 Å (cis-O (O_c_)) are shorter than the corresponding Mo–O bonds of cis-MoO_2_(acac)_2_, by 0.014 and 0.029 Å, respectively, whereas, in contrast, the W = O bonds of the WO_2_ unit of 1.705 Å are 0.013 Å longer than the Mo = O bonds of the MoO_2_ fragment. The calculated WO bond lengths compare well with the averaged, slightly shorter ones of a reported X-ray structure (i.e., 1.980 (W–O_t_), 2.168 (W–O_c_), and 1.715 Å (W = O)).^64^ Shorter bonds between the metal and its acac ligands may reflect a more tightly bonded complex and therefore suggest higher barriers for racemization due to enhanced friction and this is indeed the case.

**Figure 3:**
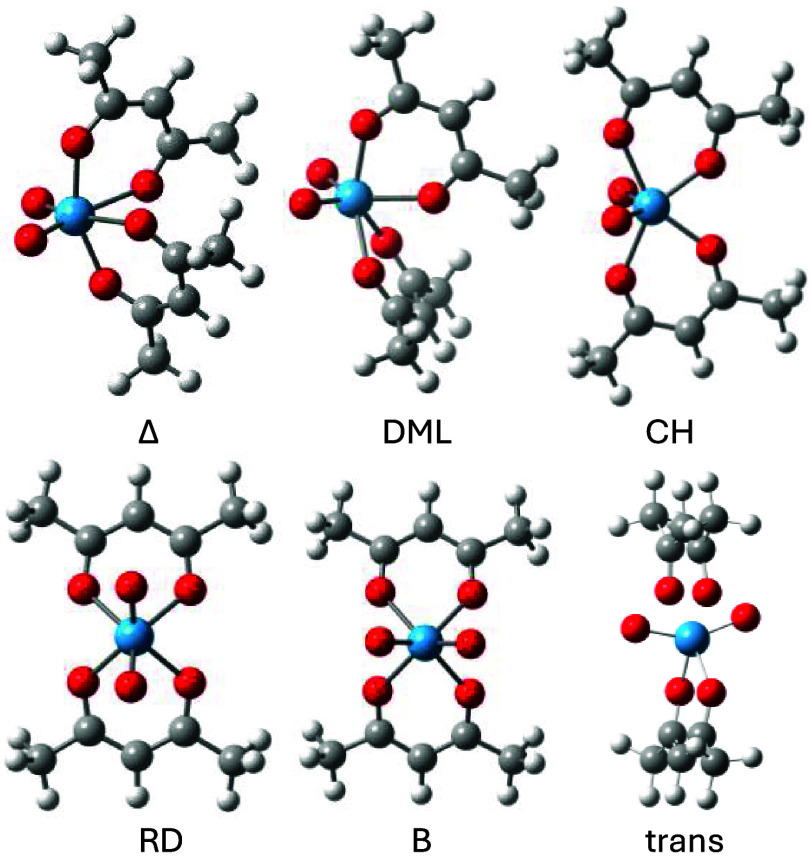
WO_2_(acac)_2_ structures at ωB97X-D/6–31G­(d) and LANL2DZ for W.

### Racemizing cis-WO_2_(acac)_2_

Four transition structures were identified for racemizing WO_2_(acac)_2_. These are the Bailar (B), Ray-Dutt (RD), Conte-Hippler (CH), and Dhimba-Muller-Lammertsma (DML) twists in accordance with Muetterties’ topological graph (Figure 2). The transition structures are shown in Figure 3 and their relative energies are given in Table 1 together with those of MoO_2_(acac)_2_ for comparison. An important observation is that the new CH and DML twists are equally prominent for WO_2_(acac)_2_ in that their racemization barriers are a sizable 9.3–13.1 kcal/mol lower in energy than the established RD and B twists. The most favored racemization path is the DML twist of 21.9 kcal/mol, which is 4.5 kcal/mol more demanding than the one for MoO_2_(acac)_2_ and in line with tungsten’s slightly tighter arrangement of its chelates. The next best racemization path is by means of the 2.6 kcal/mol less favorable CH twist. Both the RD and B twists of about 34–35 kcal/mol are unlikely at room temperature. All of this illustrates that changing the metal center of MO_2_(acac)_2_ from Mo to W increases the racemization barrier for each pathway by ca. 5–7 kcal/mol and by 2 kcal/mol for the high-energy trans form (Table 1).

**Table 1:** MO_2_(acac)_2_ Relative Energies (in kcal/mol) at ωB97X-D/6-311+G­(2d,p) and LANL2DZ for W and Mo of Minima (Δ) and Transition Structures.

	Δ	RD	B	CH	DML	trans
W	0.0	34.0	35.5	24.5	21.9	52.9
Mo^42^	0.0	27.5	28.7	20.0	17.4	50.6

The higher racemization barriers of WO_2_(acac)_2_ compared to MoO_2_(acac)_2_ are likely due to the shorter distances between tungsten and the carbonyl oxygens of the acac ligands. For instance, these M–O interactions for the favored DML transition structure (C_s_ symm.) of 2.064, 2.132, and 2.163 Å are respectively 0.044, −0.012, and 0.014 Å shorter than in the molybdenum complex. Likewise, for the CH twist structure (C_2v_ symm.), the W–O distances of 2.021 and 2.230 Å are shorter than the Mo–O ones by 0.019 and 0.034 Å, respectively. For the RD and B transition structures, the M–O interactions of 2.114 and 2.132 Å are equally shorter for WO_2_(acac)_2_ by 0.015 Å. The pronounced, but uneven shortening of the W–O bonds versus the Mo–O bonds in the CH and DML twist structures may reflect their different degrees of friction.

With NMR spectroscopy, Conte and Hippler determined a barrier (*E*_a_) of 16.9 kcal/mol for racemizing cis-MoO_2_(acac)_2_ in benzene. This barrier agrees remarkably well with the DML twist of 16.3 kcal/mol when solvation effects (benzene) are included. Because of the energy-lowering effect of solvation (17.4 → 16.3 kcal/mol), it seems to us that the higher barrier of 21.9 kcal/mol for racemizing WO_2_(acac)_2_ may still be too modest for making practical use of Δ and Λ enantiomers in catalysis.

### Racemizing cis-WO_2_(nacnac)_2_

Our next step is to explore two β-diketiminate (nacnac) ligands, which can each be substituted at the N-sites for a total of four, to induce steric hindrance that increases the barrier for racemization. The added advantage is that WO_2_(nacnac)_2_ can be directly compared with our recent MoO_2_(nacnac)_2_ study. Because of the relevance of solvation, we include this effect at a larger basis for acetonitrile, which is a common solvent in catalytic processes.

The W-complex with simple nacnac instead of acac ligands is, in fact, a more relaxed structure because the W–N bonds are slightly longer than the W–O bonds. The first question is whether WO_2_(nacnac)_2_ has the same four racemization channels as WO_2_(acac)_2_ and its molybdenum homologue. This is indeed the case. The structures are displayed in Figure 4 and the relative energies are given in Table 2.

**Table 2:** MO_2_(nacnac)_2_ Relative Energies (in kcal/mol) at ωB97X-D/6-311+G­(2d,p) and LANL2DZ for W and Mo of Minima (Δ) and Transition Structures with MeCN Solvation

	Δ	RD	B	CH	DML	trans
W	0.0	32.6	18.8	20.2	9.8	45.0
Mo^43^	0.0	26.6	14.9	15.7	6.9	45.6

**Figure 4:**
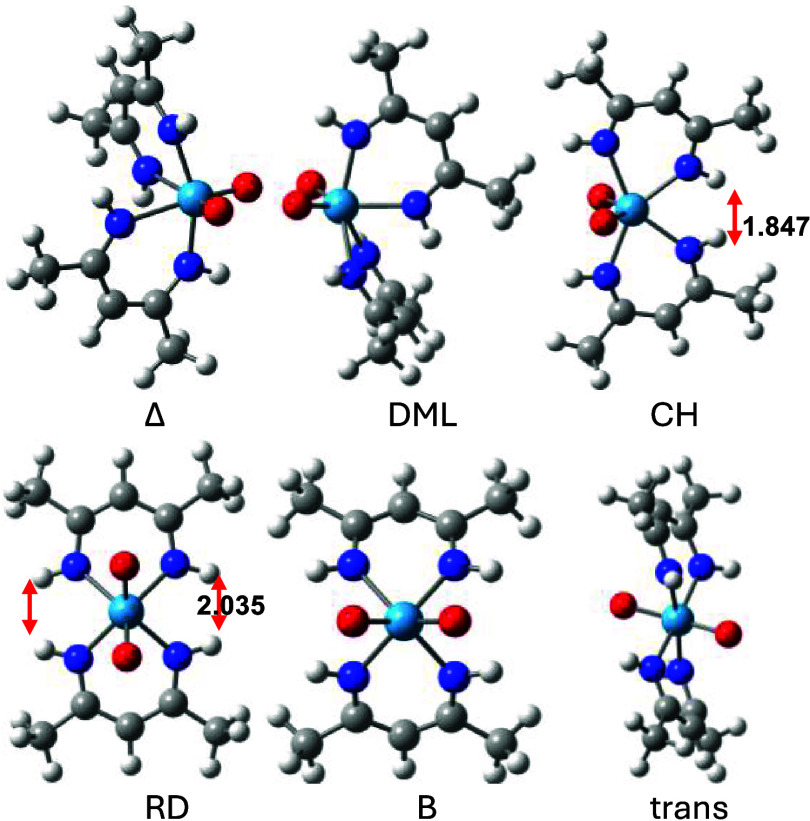
WO_2_(nacnac)_2_ structures at ωB97X-D/6–31G­(d) and LANL2DZ for W. Double-headed red arrows for CH and RD indicate close distances (in Å).

The relative energies of the transition structures differ markedly from those of WO_2_(acac)_2_ (Table 1), but mimic MoO_2_(nacnac)_2_ (Table 2), which has 3–6 kcal/mol lower barriers for each of the racemization pathways because of the longer Mo–N bonds. Exchanging tungsten’s acac ligands for nacnac ligands has a striking effect on the racemization barriers. It reduces the B and DML twist barriers by as much as 16.7 and 12.1 kcal/mol to 18.8 and 9.8 kcal/mol, respectively. The 4.3 kcal/mol reduction for the CH twist to 20.2 kcal/mol is more modest, whereas the barrier for the RD twist reduces by only 1.4 to 32.6 kcal/mol. These differences in relative energies are reflected in the WO_2_(nacnac)_2_ geometries as the W–N bonds are longer than the W–O bonds by up to 0.09 Å. For example, the CH twist structure changes from C_s_ to C_2_ symmetry to minimize the NH···HN interaction to a still very short 1.847 Å (Figure 4). As a consequence, the CH twist structure exists as an enantiomeric Δ and Λ pair (see also Figure 2). The 2.035 Å short NH···HN in the RD twist structure (Figure 4) causes the angle between the two ligand planes to increase to 167.2°, which is 7.1° more than in WO_2_(acac)_2_. In contrast, this angle between the ligands of the B twist structure reduces from 133.7° (acac) to 101.5° (nacnac) and shows normal NH···HN interactions of 2.596 Å, which underlies its 16.7 kcal/mol reduced barrier. The 12.1 kcal/mol reduced DML barrier can be partly attributed to the favorable NH···N interactions of one NH group hanging over both N atoms of the other nacnac ligand.

The conclusion of this section is that (a) all four racemization pathways are present, (b) replacing acac for nacnac ligands reduces some of the racemization barriers significantly, (c) all barriers are ∼3 kcal/mol are higher than those of MoO_2_(nacnac)_2_, and (d) the most favorable racemization pathway is by the DML twist requiring merely 9.8 kcal/mol. The obvious next step is to explore the effects of N-substitution of the ligands in the hope that steric effects contribute to raising the barriers.

### Racemizing cis-WO_2_(nacnac)_2_^Me4^

We start by Me-substituting both N-sites of the two β-diketiminate chelates. For this far more congested system, all four transition structures for racemization were again identified, as well as the trans isomer. The geometries of these WO_2_(nacnac)_2_^Me4^ structures are shown in Figure 5 and the relative energies listed in Table 3, together with those of the Mo analogue.

**Table 3:** Relative Energies (in kcal/mol) of Tetra N–Me Substituted MO_2_(nacnac)_2_ at ωB97X-D/6-311+G­(2d,p) and LANL2DZ for W and Mo of Minima (Δ) and Transition Structures with MeCN Solvation

	Δ	RD	B	CH	DML	trans
W	0.0	45.6	34.5	36.3	24.0	34.5
Mo^43^	0.0	37.8	28.5	30.5	25.1	34.7

**Figure 5:**
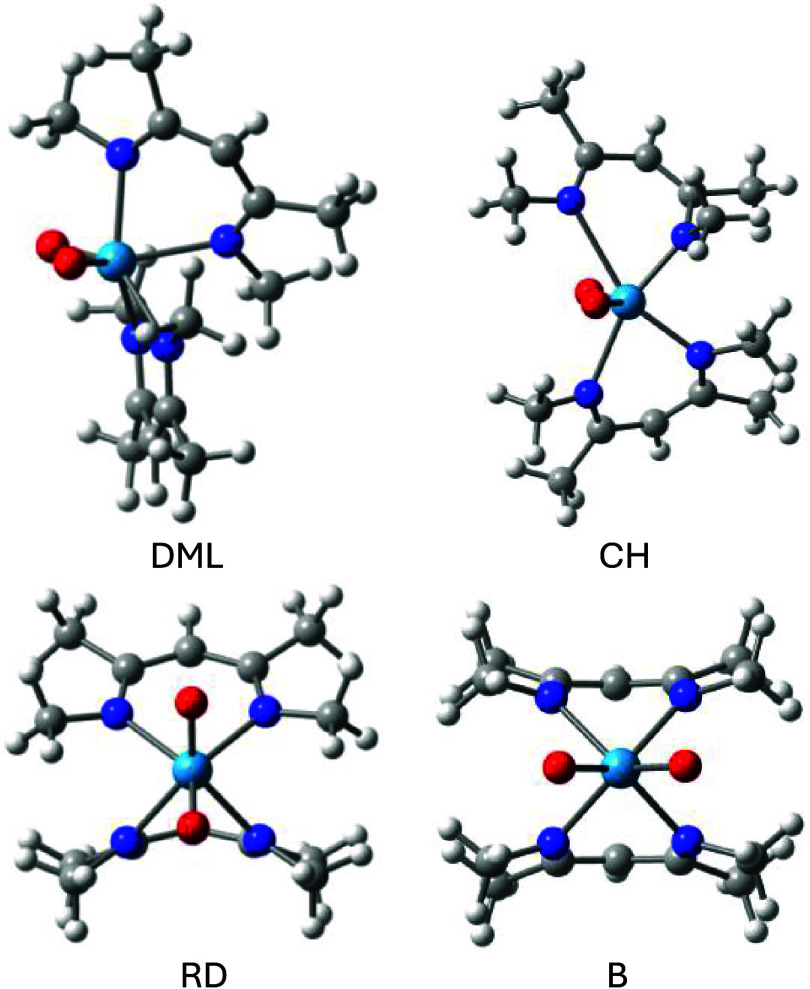
WO_2_(nacnac)_2_^Me4^ transition structures at ωB97X-D/6–31G­(d) and LANL2DZ for W.

Both the geometries and their relative energies reveal the impact of N–Me substitution. The influence is already evident for Δ-cis-WO_2_(nacnac)_2_^Me4^ (C_2_ symm.) as the angle between its nacnac planes reduces from 84.9° for the N-unsubstituted complex to only 29.3°, with a 0.7 Å increase of the out-of-plane distance of the tungsten atom to 0.852 Å. Moreover, the angle between the ligand planes and that of WO_2_ reduces by 21.5 to 56.6° as shown in the space filling views in Figure 6 (top).

**Figure 6:**
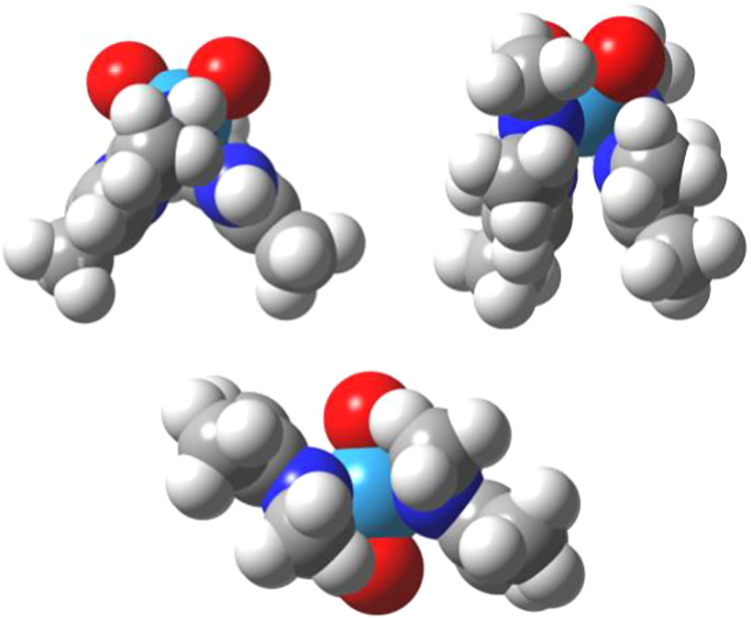
Space filling views of Δ-cis-WO_2_(nacnac)_2_ (top left), Δ-cis-WO_2_(nacnac)_2_^Me4^ (top right), and the trans isomer (bottom).

Revealing are the relative energies of the transition structures. Whereas the favored racemization pathway is again the DML twist, its 24.0 kcal/mol barrier reflects a 14.2 kcal/mol increase over the N-unsubstituted WO_2_(nacnac)_2_ (C_s_ symm.). The reason for this large effect is that the N-Me group eliminates stabilization of a N–H group of one ligand hanging over both N atoms of the other ligand (Figure 3). Instead, the N-Me group causes the ligand plane to rotate 32 ° from orthogonality (Figure 5).

Equally striking are the still 10.5 kcal/mol higher barriers for both the trans isomer (C_2h_ symm.) and the B twist of 34.5 kcal/mol. Avoided clashing of its N-Me substituents causes nonplanarity of the trans isomer (Figure 6, bottom); its parallel ligand planes are tilted by 141.4° from the central WN_4_ plane. The B twist transition structure (C_2v_ symm., Figure 5) has a 27.2° angle between its ligand planes (defined by C_2_N_2_) and 67.1° between its WN_2_ planes to minimize N-Me steric repulsion (d­(NH···HN) = 2.297 Å); in contrast, the N-unsubstituted structure has unbent ligands. The CH twist structure is even 1.8 kcal/mol less favored than both the B twist and trans structures, which reflects a relative increase by a large 16.1 kcal/mol over the N-unsubstituted form. The N-Me groups cause the CH structure to show some resemblance with the trans structure, but its ligands point instead in the same direction (∠99.8°; shortest NH···HN = 2.300 Å). By far the least favored WO_2_(nacnac)_2_^Me4^ transition structure is the RD twist (ΔΔE = 45.6 kcal/mol) because both N-Me groups of one ligand clash with those of the other, thereby causing significant distortion.

The conclusions of this section are that on N-Me substitution of both nacnac ligands (a) all four twists are still present, (b) the B, RD, and CH twist barriers increase by 6–8 kcal/mol and the DML twist by 14 kcal/mol, (c) and that the DML twist is the only viable racemization pathway requiring 24.0 kcal/mol, which bodes well for experimental pursuit.

### Racemizing cis-WO_2_(nacnac)_2_^Ph4^

We also looked at substituting the β-diketiminate ligands with *N*-phenyl groups. The relative energies of the various twist barriers, except for the trans isomer, are given in Table 4 together with those of MoO_2_(nacnac)_2_^Ph4^. The chiral minimum energy Δ and the DML and CH twist structures are shown in Figure 7.

**Table 4:** Relative Energies (in kcal/mol) of Tetra N–Ph Substituted MO_2_(nacnac)_2_ at ωB97X-D/6-311+G­(2d,p) and LANL2DZ for W and Mo of Minima (Δ) and Transition Structures with MeCN Solvation

	Δ	RD	B	CH	DML
W	0.0	43.1	39.0	27.2	25.5
Mo	0.0	36.2	33.3	22.1	20.3

**Figure 7:**
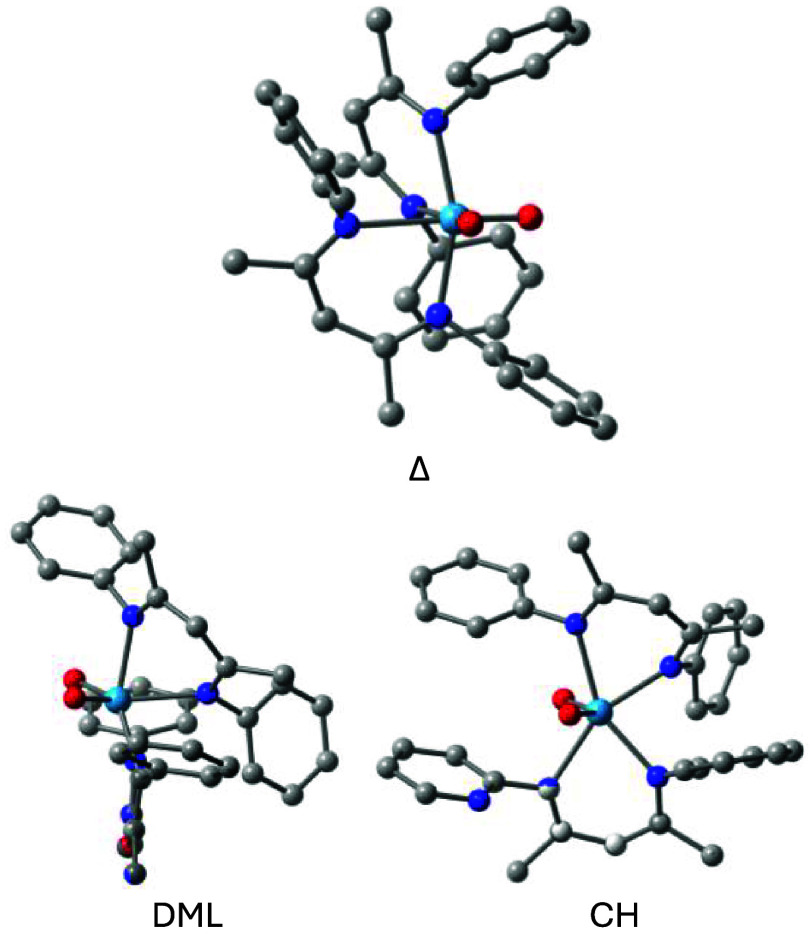
WO_2_(nacnac)_2_^Me4^ structures for Δ, DML, and CH at ωB97X-D/6–31G­(d) and LANL2DZ for W. Hydrogen atoms are omitted for clarity.

Except for the CH twist structure, the trends in relative energies of the transition structures mimic those found for the N-Me tetrasubstituted complex. The preferred WO_2_(nacnac)_2_^Ph4^ isomerization path is again by means of the DML twist with a barrier of 25.5 kcal/mol, which is slightly, but distinctly higher (by 1.5 kcal/mol) than the all N-Me derivative. Whereas the CH twist of 27.2 kcal/mol is less favorable, its relative energy has decreased from WO_2_(nacnac)_2_^Me4^ by 9.1 kcal/mol because of the reduced steric repulsion between the N-substituents. The B and RD twists have such high relative energies that they are unlikely to be relevant for racemization. Finally, all the twists (B, RD, CH, and DML) have barriers that are >5 kcal/mol higher than those of the Mo homologue, which again is a reflection of the more contracted W-complex.

This section shows WO_2_(nacnac)_2_^Ph4^ racemization to occur preferentially via a DML twist with a sizable barrier of 25.5 kcal/mol (unsolvated 27.4 kcal/mol). Using its projected Δ*G* (GoodVibes v3.2)^76,77^ of 25.7 kcal/mol and Eyring’s equation suggests a half-life of ca. 9 days (25 °C) for the chiral structure. Of course, the chiral integrity may be further enhanced by functionalizing the phenyl groups with substituents and using a less stabilizing solvent.

## Conclusions

This computational study reveals the DML twist to have the lowest barrier of the four racemization pathways for both WO_2_(acac)_2_ and WO_2_(nacnac)_2_. This DML twist barrier is lower than that for the CH twist and much lower than those for the B and RD twists. Increasing steric congestion by N-Me and N-Ph substitution of the two nacnac chelates raises all four (B, RD, CH, DML) barriers for racemization, substantially, of which the DML twist remains distinctly the preferred pathway. The magnitude of the barrier for the established B twist and particularly the RD twist makes these unlikely to occur in the tungsten complexes. The DML twist barrier for WO_2_(acac)_2_ amounts to 22 kcal/mol and to 10 kcal/mol for the more relaxed WO_2_(nacnac)_2_ when the effects of solvation are included. N-Me substitution has a large geometrical impact and increases the racemization barrier to 24 kcal/mol and to 27 kcal/mol when all N-sites are substituted instead with phenyl groups. This barrier can presumably be further increased by appropriate substitution of the phenyl groups.

This extensive study of WO_2_(chel)_2_ suggests that chiral-at-tungsten complexes are viable targets for experimental asymmetric catalysis.

## Supplementary Material



## Notes

Absolute (in au) and relative energies (in kcal/mol) and lowest (imaginary) frequencies (in cm^–1^) of the WO_2_(acac)_2_ minimum (Δ) and transition structures (Table S1); absolute (in au) and relative energies (in kcal/mol) and lowest (imaginary) frequencies in (cm^–1^) of the MoO_2_(acac)_2_ minimum (Δ) and transition structures (Table S2); absolute (in au) and relative energies (in kcal/mol) and lowest (imaginary) frequencies in (cm^–1^) of the minimum (Δ) and transition structures for WO_2_(nacnac)_2_ (abbreviated as WO_2_NN_2_) and its tetra N-Me and tetra N-Ph derivatives (Table S3); absolute (in au) and relative energies (in kcal/mol) and lowest (imaginary) frequencies in (cm^–1^) of the minimum (Δ) and transition structures for MoO_2_(nacnac)_2_ (abbreviated as MoO_2_NN_2_) and its tetra N-Me and tetra N-Ph derivatives (Table S4); and the geometrical parameters of all optimized tunsten structures (PDF)
