# Supplementary material for: Chiral-at-Tungsten Dioxo Complexes—A Computational Study on Inhibiting Racemization
Source: Inorg Chem. 2025 May 29;64(27):13569–76. doi: 10.1021/acs.inorgchem.5c01280 (PMC12264964; doi:10.1021/acs.inorgchem.5c01280)
Supplement: Supplementary file 1 [file ic5c01280_si_001.pdf]

Supporting Information

Chiral-at-tungsten dioxo complexes - A computational study on inhibiting racemization

George Dhimba,[a] Alfred Muller,[a] and Koop Lammertsma[a,b,*]

[a] Department of Chemical Sciences, University of Johannesburg, Auckland Park, Johannesburg, 2006, South Africa

[b] Department of Chemistry and Pharmaceutical Sciences, Faculty of Sciences, Vrije Universiteit Amsterdam, De Boelelaan 1108, 1081 HZ Amsterdam, The Netherlands.

Email address: k.lammertsma@vu.nl

ENERGIES

Table S1. Absolute (in au) and relative energies (in kcal/mol) and lowest (imaginary) frequencies (in cm^{-1}) of the $WO_2(acac)_2$ minimum (Δ) and transition structures.

	6-311+G(2d,p) + LANLDZ for W		6-31G(d,p) + LANLDZ for W				
**$WO_2(acac)_2$**	***E(a.u)***	***Δ***	***E(a.u)***	***Δ***	***G(a.u)***	***Δ***	***ν***
Λ	-908.83387	0.0	-908.56649	0.0	-908.38091	0.0	0
Ray_Dutt	-908.77962	34.0	-908.51242	33.9	-908.32298	36.4	-140
Bailer	-908.77735	35.5	-908.51125	34.7	-908.32476	35.2	-142
C-H	-908.79476	24.5	-908.52725	24.6	-908.34146	24.8	-24
DML	-908.79904	21.9	-908.53206	21.6	-908.34569	22.1	-100
trans	-908.74953	52.9	-908.48105	53.6	-908.30734	46.2	-54

Table S2. Absolute (in au) and relative energies (in kcal/mol) and lowest (imaginary) frequencies in (cm^{-1}) of the $MoO_2(acac)_2$ minimum (Δ) and transition structures.

	6-311+G(2d,p) + LANLDZ for W		6-31G(d,p) + LANLDZ for W				
**$MoO_2(acac)_2$**	***E(a.u)***	***Δ***	***E(a.u)***	***Δ***	***G(a.u)***	***Δ***	***ν***
Λ	-908.47328	0.0	-908.20621	0.0	-908.02015	0.0	0
Ray_Dutt	-908.42951	27.5	-908.16250	27.4	-907.97327	29.4	-109
Bailer	-908.42756	28.7	-908.16219	27.6	-907.97515	28.2	-106
C-H	-908.44143	20.0	-908.17447	19.9	-907.98932	19.3	-24
DML	-908.44555	17.4	-908.17949	16.8	-907.99303	17.0	-85
trans	-908.39258	50.6	-908.12513	50.9	-907.93901	50.9	-17

Table S3. Absolute (in au) and relative energies (in kcal/mol) and lowest (imaginary) frequencies in (cm^{-1}) of the minimum (Δ) and transition structures for $WO_2(nacnac)_2$ (abbreviated as WO_2NN_2) and its tetra N-Me and tetra N-Ph derivatives.

	6-311+G(2d,p) + LANLDZ for W + solvent[a]				6-31G(d,p) + LANLDZ for W				
**WO_2NN_2**	***E(a.u)^{MeCN}***	***Δ***	***$\Delta G_{est.}$***	***Δ***	***E(a.u)***	***Δ***	***G(a.u)***	***Δ***	***v***
Λ	-829.35871	0.0	-829.11743	0.0	-829.08898	0.0	-828.85172	0.0	21
Ray_Dutt	-829.30678	32.6	-829.06438	33.3	-829.02642	39.3	-828.78697	40.6	-129
Bailer	-829.32868	18.8	-829.08381	21.1	-829.05768	19.6	-828.81542	22.8	-93
C-H	-829.32659	20.2	-829.08280	21.7	-829.05560	20.9	-828.81459	23.3	-32
DML	-829.34307	9.8	-829.09889	11.6	-829.07555	8.4	-828.83451	10.8	-67
trans	-829.28695	45.0	-829.04597	44.8	-829.00376	53.5	-828.76562	54.0	-139
**$WO_2NN_2^{Me4}$**	***E(a.u)^{MeCN}***	***Δ***	***$\Delta G_{est.}$***	***Δ***	***E(a.u)***	***Δ***	***G(a.u)***	***Δ***	***v***
Λ	-986.55750	0.0	-986.20323	0.0	-986.26155	0.0	-985.90895	0.0	64
Ray_Dutt	-986.48484	45.6	-986.13275	44.2	-986.18183	50.0	-985.83204	48.3	-108
Bailer	-986.50257	34.5	-986.14875	34.2	-986.20586	34.9	-985.85532	33.7	-85
C-H	-986.49965	36.3	-986.15027	33.2	-986.20417	36.0	-985.85789	32.0	-57
DML	-986.51924	24.0	-986.16297	25.3	-986.22281	24.3	-985.86834	25.5	-53
trans	-986.50255	34.5	-986.15126	32.6	-986.19529	41.6	-985.846458	39.2	-162
**$WO_2NN_2^{Ph4}$**	***E(a.u)^{MeCN}***	***Δ***	***$\Delta G_{est.}$***	***Δ***	***E(a.u)***	***Δ***	***G(a.u)***	***Δ***	***v***
Λ	-1753.45767	0.0	-1752.90424	0.0	-1752.96419	0.0	-1752.41751	0.0	25
Ray_Dutt	-1753.38900	43.1	-1752.83474	43.6	-1752.88736	48.2	-1752.33923	49.1	-56
Bailer	-1753.39558	39.0	-1752.84266	38.6	-1752.89555	43.1	-1752.34989	42.4	-45
C-H	-1753.41434	27.2	-1752.86156	26.8	-1752.91971	27.9	-1752.37459	26.9	-17
DML	-1753.41703	25.5	-1752.86325	25.7	-1752.92086	27.2	-1752.37388	27.4	-60

[a] The effects of acetonitrile solvation with the PCM model are included for the larger basis set. The corresponding free energies (absolute and relative) are obtained with the aid of GoodVibes.

Table S4. Absolute (in au) and relative energies (in kcal/mol) and lowest (imaginary) frequencies in (cm^{-1}) of the minimum (Δ) and transition structures for $MoO_2(nacnac)_2$ (abbreviated as MoO_2NN_2)and its tetra N-Me and tetra N-Ph derivatives.

	6311+G(2d,p) + LANLDZ for W + solvent[a]				6-31G(d,p) + LANLDZ for W				
**MoO_2NN_2**	***E(a.u)^{MeCN}***	***Δ***	***$ΔG_{est.}$***	***Δ***	***E(a.u)***	***Δ***	***G(a.u)***	***Δ***	***ν***
Λ	-828.98978	0.0	-828.74902	0.0	-828.72516	0.0	-828.48867	0.0	23
Ray_Dutt	-828.94736	26.6	-828.70470	27.8	-828.67412	32.0	-828.43419	34.2	-103
Bailer	-828.96598	14.9	-828.72191	17.0	-828.70069	15.4	-828.45926	18.5	-78
C-H	-828.96473	15.7	-828.72129	17.4	-828.69855	16.7	-828.45791	19.3	-26
DML	-828.97873	6.9	-828.73472	9.0	-828.71612	5.7	-828.47511	8.5	-61
trans	-828.91718	45.6	-828.67651	45.5	-828.63864	54.3	-828.40106	55.0	-139
**$MoO_2NN_2^{Me4}$**	***E(a.u)^{MeCN}***	***Δ***			***E(a.u)***	***Δ***	***G(a.u)***	***Δ***	***ν***
Λ	-986.18767	0.0	-985.83545	0.0	-985.89635	0.0	-985.54597	0.0	61
Ray_Dutt	-986.12739	37.8	-985.77524	37.8	-985.83014	41.5	-985.48025	41.2	-84
Bailer	-986.14233	28.5	-985.78848	29.5	-985.85063	28.7	-985.49993	28.9	-71
C-H	-986.13907	30.5	-985.78975	28.7	-985.84704	30.9	-985.50072	28.4	-65
DML	-986.14761	25.1	-985.79420	25.9	-985.85735	24.5	-985.50628	24.9	-53
trans	-986.13241	34.7	-985.78206	33.5	-985.82867	42.5	-985.48119	40.6	-229
**$MoO_2NN_2^{Ph4}$**	***E(a.u)^{MeCN}***	***Δ***			***E(a.u)***	***Δ***	***G(a.u)***	***Δ***	***ν***
Λ	-1753.08842	0.0	-1752.53602	0.0	-1752.59875	0.0	-1752.05339	0.0	24
Ray_Dutt	-1753.03072	36.2	-1752.47661	37.3	-1752.53436	40.4	-1751.98632	42.1	-35
Bailer	-1753.03540	33.3	-1752.48347	33.0	-1752.54055	36.5	-1751.99612	35.9	-25
C-H	-1753.05318	22.1	-1752.50039	22.4	-1752.56253	22.7	-1752.01742	22.6	-12
DML	-1753.05601	20.3	-1752.50250	21.0	-1752.56321	22.3	-1752.01665	23.1	-53

[a] The effects of acetonitrile solvation with the PCM model are included for the larger basis set. The corresponding free energies (absolute and relative) are obtained with the aid of GoodVibes.

Geometries

Cis-Δ $WO_2(acac)_2$

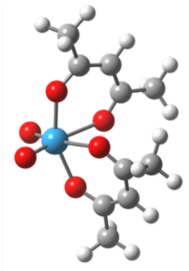

Atom	X	Y	Z
W	0.00007	1.14722	0.00056
O	1.90313	0.72386	0.46730
O	0.30493	-0.63213	-1.29864
O	-1.90312	0.72436	-0.46626
O	-0.30527	-0.63228	1.29933
O	0.27261	2.19025	-1.32057
O	-0.27223	2.19023	1.32174
C	4.02778	-0.12355	0.94107
C	2.71675	-0.25113	0.22175
C	2.45741	-1.31744	-0.61655
C	1.26187	-1.44470	-1.36317
C	1.09351	-2.59671	-2.31914
H	4.50251	0.82048	0.65509
H	3.84082	-0.07955	2.01871
H	4.70308	-0.95217	0.71980
H	3.22612	-2.06979	-0.73891
H	1.89095	-3.33763	-2.23188
H	0.12441	-3.07134	-2.13960
H	1.07995	-2.20512	-3.34191
C	-1.26211	-1.44505	1.36319
C	-2.71663	-0.25093	-0.22161
C	-1.09398	-2.59731	2.31889
C	-2.45737	-1.31769	0.61616
C	-4.02745	-0.12316	-0.94127
H	-1.08115	-2.20605	3.34179
H	-1.89116	-3.33842	2.23091
H	-0.12464	-3.07161	2.13977
H	-3.22600	-2.07023	0.73787
H	-4.70273	-0.95196	-0.72058
H	-4.50237	0.82068	-0.65502
H	-3.84016	-0.07867	-2.01884

DML-$WO_2(acac)_2$

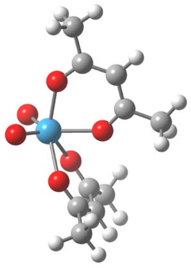

Atom	X	Y	Z
W	0.07138	0.15547	1.45860
O	0.83611	0.15626	-0.53111
O	2.11266	0.24219	1.75211
O	-1.20783	-1.22655	0.39457
O	-1.31096	1.40189	0.35403
O	0.00269	-1.14579	2.55229
O	-0.10316	1.47893	2.51310
C	1.97388	0.18415	-2.58273
C	1.98410	0.20313	-1.08056
C	3.17689	0.26707	-0.36081
C	3.16815	0.28146	1.03205
C	4.44392	0.34593	1.82083
H	1.47121	-0.72641	-2.92385
H	1.38645	1.03363	-2.94529
H	2.97878	0.22733	-3.00716
H	4.12159	0.30402	-0.88673
H	5.32541	0.37706	1.17762
H	4.42328	1.23426	2.45974
H	4.49990	-0.52631	2.47947
C	-2.07214	1.25250	-0.65405
C	-1.97668	-1.16801	-0.61732
C	-2.62900	2.53117	-1.22180
C	-2.41339	0.01815	-1.20643
C	-2.42926	-2.50322	-1.14618
H	-1.80094	3.19295	-1.49449
H	-3.26270	2.35929	-2.09468
H	-3.20843	3.04221	-0.44642
H	-3.08566	-0.02126	-2.05394
H	-3.07522	-2.40824	-2.02184
H	-1.55071	-3.10371	-1.40233
H	-2.96503	-3.03669	-0.35471

CH-$WO_2(acac)_2$

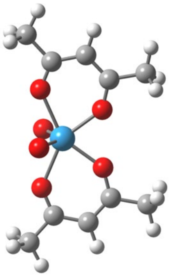

Atom	X	Y	Z
W	-0.00029	-0.41939	0.04845
O	-2.05462	-1.28506	0.10762
O	-1.20907	1.19657	-0.06561
O	2.05338	-1.28639	0.11079
O	1.20971	1.19577	-0.06409
O	0.00046	-1.38068	-1.36685
O	-0.00169	-1.17514	1.58355
C	-3.49065	0.57517	-0.01873
H	-4.51832	0.91340	-0.04072
C	3.49081	0.57285	-0.01416
H	4.51873	0.91038	-0.03489
C	-4.33859	-1.81035	0.15662
H	-5.31994	-1.33950	0.06705
H	-4.27494	-2.33906	1.11296
H	-4.21229	-2.55361	-0.63593
C	4.33693	-1.81317	0.16310
H	4.21082	-2.55708	-0.62887
H	4.27207	-2.34094	1.11988
H	5.31868	-1.34307	0.07399
C	2.73830	2.96563	-0.18495
H	2.27371	3.35059	-1.09843
H	3.80617	3.19275	-0.19647
H	2.26588	3.47563	0.66071
C	-2.73632	2.96747	-0.18809
H	-2.26463	3.47700	0.65826
H	-3.80402	3.19531	-0.20093
H	-2.27032	3.35229	-1.10090
C	-3.21260	-0.81351	0.07773
C	-2.47467	1.49312	-0.08453
C	2.47553	1.49147	-0.08145
C	3.21171	-0.81562	0.08232

RD-$WO_2(acac)_2$

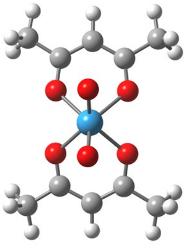

Atom	X	Y	Z
W	0.00002	-0.00002	0.72501
O	-1.20285	-1.38655	-0.32304
O	-1.20297	1.38644	-0.32299
O	1.20286	1.38655	-0.32302
O	1.20298	-1.38645	-0.32307
O	1.28511	0.00001	1.85237
O	-1.28504	-0.00010	1.85240
C	-3.20901	-2.51335	-0.78326
C	-2.45057	-1.23536	-0.55775
C	-3.10098	-0.00013	-0.64492
C	-2.45068	1.23515	-0.55771
C	-3.20923	2.51309	-0.78317
H	-2.68020	-3.12249	-1.52206
H	-3.22291	-3.07752	0.15560
H	-4.23546	-2.34091	-1.11282
H	-4.15825	-0.00017	-0.87936
H	-4.23565	2.34058	-1.11275
H	-3.22318	3.07722	0.15572
H	-2.68046	3.12231	-1.52194
C	2.45068	-1.23515	-0.55782
C	2.45057	1.23536	-0.55777
C	3.20922	-2.51309	-0.78334
C	3.10098	0.00013	-0.64501
C	3.20901	2.51336	-0.78326
H	2.68043	-3.12228	-1.52212
H	4.23564	-2.34056	-1.11296
H	3.22321	-3.07724	0.15553
H	4.15824	0.00018	-0.87948
H	4.23544	2.34093	-1.11287
H	2.68017	3.12253	-1.52202
H	3.22294	3.07749	0.15562

B-$WO_2(acac)_2$

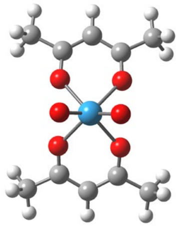

Atom	X	Y	Z
W	0.00000	0.00000	0.84818
O	1.37954	1.28141	-0.15171
O	1.37952	-1.28143	-0.15170
O	-1.37954	-1.28141	-0.15170
O	-1.37952	1.28143	-0.15171
O	-0.00001	-1.26356	1.98699
O	0.00001	1.26356	1.98699
C	3.18330	2.52608	-0.99263
C	2.54932	1.20633	-0.65202
C	3.20690	-0.00003	-0.89047
C	2.54930	-1.20638	-0.65201
C	3.18326	-2.52614	-0.99262
H	2.52560	3.07469	-1.67393
H	3.27286	3.12221	-0.07897
H	4.16771	2.40821	-1.45054
H	4.20533	-0.00004	-1.30779
H	4.16767	-2.40828	-1.45053
H	3.27281	-3.12226	-0.07896
H	2.52555	-3.07474	-1.67392
C	-2.54930	1.20637	-0.65201
C	-2.54932	-1.20634	-0.65201
C	-3.18326	2.52613	-0.99263
C	-3.20690	0.00002	-0.89047
C	-3.18330	-2.52608	-0.99262
H	-2.52555	3.07473	-1.67393
H	-4.16767	2.40827	-1.45054
H	-3.27281	3.12226	-0.07897
H	-4.20533	0.00003	-1.30778
H	-4.16771	-2.40821	-1.45053
H	-2.52560	-3.07470	-1.67392
H	-3.27286	-3.12221	-0.07895

trans-$WO_2(acac)_2$

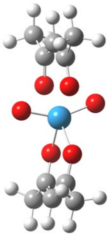

Atom	X	Y	Z
W	0.27650	0.00016	0.05313
O	0.14067	1.56292	-1.31034
O	0.14202	-1.56274	-1.31029
O	0.40619	-1.31508	1.58465
O	0.40505	1.31548	1.58468
O	-1.45575	-0.00057	-0.23080
O	1.91034	0.00088	-0.57904
C	0.00000	0.00000	-3.14228
H	-0.08500	-0.00005	-4.22162
C	0.00000	0.00000	3.49852
H	-0.18123	-0.00009	4.56493
C	0.04607	-1.27834	-2.55252
C	0.04497	1.27840	-2.55255
C	0.16154	1.22111	2.84012
C	0.16259	-1.22095	2.84009
C	-0.02548	2.49221	-3.44279
H	-0.12577	2.23547	-4.49875
H	-0.87646	3.10825	-3.13632
H	0.88118	3.08851	-3.29960
C	-0.02340	-2.49224	-3.44270
H	-0.87412	-3.10875	-3.13646
H	-0.12355	-2.23561	-4.49871
H	0.88355	-3.08801	-3.29921
C	0.09752	-2.52657	3.57016
H	1.03564	-3.06887	3.41586
H	-0.08001	-2.39452	4.63873
H	-0.70412	-3.13327	3.13680
C	0.09537	2.52665	3.57022
H	-0.08208	2.39442	4.63879
H	1.03304	3.06973	3.41596
H	-0.70676	3.13269	3.13687

Cis-Δ $WO_2(nacnac)_2$

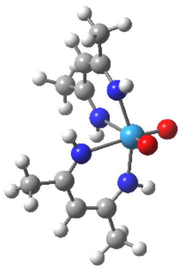

Atom	X	Y	Z
O	-0.13989	1.95566	-1.35068
C	2.95084	-0.32047	-0.19104
C	2.83836	-1.28032	0.79047
H	3.71050	-1.87830	1.02757
C	1.64100	-1.54482	1.50635
C	-1.64062	-1.53944	-1.50887
C	-2.83882	-1.27610	-0.79391
H	-3.71119	-1.87240	-1.03437
C	-2.95143	-0.32038	0.19156
O	0.13922	1.95108	1.36093
N	1.95318	0.48244	-0.60056
N	-1.95347	0.48000	0.60540
H	2.23826	1.10222	-1.35088
H	-2.23871	1.09678	1.35813
C	1.67585	-2.65908	2.52502
H	0.69356	-2.83300	2.97298
H	2.01981	-3.58974	2.06217
H	2.38189	-2.40965	3.32431
C	4.27478	-0.13162	-0.89194
H	4.15584	-0.26042	-1.97402
H	4.64845	0.88377	-0.71780
H	5.02664	-0.84090	-0.54027
C	-1.67438	-2.65244	-2.52893
H	-2.00058	-3.58781	-2.06255
H	-2.39370	-2.41149	-3.31885
H	-0.69572	-2.81362	-2.98941
C	-4.27583	-0.13345	0.89210
H	-4.15797	-0.26727	1.97369
H	-4.64842	0.88306	0.72223
H	-5.02802	-0.84045	0.53655
W	-0.00018	0.90259	0.00334
N	0.55022	-0.86301	1.30665
H	-0.23154	-1.17594	1.87466
N	-0.54962	-0.85907	-1.30565
H	0.23287	-1.17129	-1.87303

DML-$WO_2(nacnac)_2$

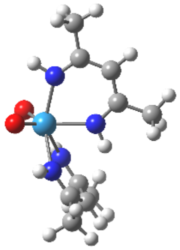

Atom	X	Y	Z
O	0.40221	-2.11609	1.04650
O	0.49143	-1.71758	-1.61146
N	-1.52794	-0.51713	1.18645
H	-1.40253	-1.03304	2.05208
N	0.32665	1.25478	0.18896
H	-0.60290	1.66931	0.21963
C	1.27865	2.15908	0.35696
C	2.63416	1.83885	0.35486
H	3.36392	2.62514	0.49763
C	3.07820	0.52933	0.17306
C	-2.50890	0.62649	-1.23626
C	-3.01103	0.95193	0.03163
H	-3.88983	1.58186	0.09659
C	-2.58986	0.26453	1.17867
C	-3.24796	1.10362	-2.46444
H	-3.78674	0.26426	-2.91870
H	-3.97591	1.88107	-2.22104
H	-2.55026	1.49696	-3.21073
C	-3.41241	0.36831	2.44158
H	-3.97152	-0.56195	2.59383
H	-2.76898	0.51900	3.31434
H	-4.13045	1.19006	2.38939
C	4.55901	0.23657	0.17892
H	5.14822	1.14229	0.33537
H	4.79981	-0.47573	0.97554
H	4.85887	-0.21181	-0.77451
C	0.84917	3.59227	0.55696
H	0.27140	3.93603	-0.30811
H	0.20560	3.67265	1.43994
H	1.70403	4.25856	0.68947
W	0.19225	-0.87404	-0.13468
N	-1.44111	-0.12898	-1.40305
H	-1.25420	-0.36996	-2.37166
N	2.26554	-0.49663	-0.00852
H	2.75749	-1.37690	-0.12425

CH-WO_2(nacnac)$_2$

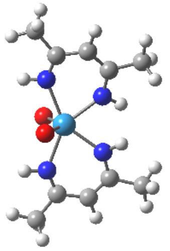

Atom	X	Y	Z
W	0.00000	-0.46495	-0.00003
O	0.14785	-1.34161	-1.48536
O	-0.14785	-1.34162	1.48529
C	-4.45653	-1.72571	-0.01477
C	-3.27257	-0.79143	0.01046
C	-3.52265	0.59467	0.20223
C	-2.54375	1.52872	-0.01310
C	-2.89930	2.99506	-0.05623
H	-4.15098	-2.76951	-0.12463
H	-5.02623	-1.62711	0.91512
H	-5.12591	-1.46584	-0.84150
H	-4.53947	0.91970	0.38535
H	-2.32648	3.55142	0.69435
H	-2.65561	3.41262	-1.04084
H	-3.96304	3.15857	0.12767
C	2.54374	1.52872	0.01304
C	3.27257	-0.79142	-0.01053
C	2.89929	2.99507	0.05617
C	3.52264	0.59468	-0.20229
C	4.45653	-1.72571	0.01470
H	3.96303	3.15858	-0.12773
H	2.32647	3.55142	-0.69440
H	2.65561	3.41262	1.04078
H	4.53946	0.91971	-0.38542
H	5.12591	-1.46583	0.84143
H	4.15099	-2.76950	0.12456
H	5.02623	-1.62711	-0.91519
N	1.24068	1.23724	0.21880
H	0.73796	2.05033	0.55483
N	-1.24069	1.23724	-0.21886
H	-0.73796	2.05033	-0.55489
N	2.07361	-1.24000	0.19179
H	2.02718	-2.23408	0.39056
N	-2.07361	-1.24000	-0.19185
H	-2.02718	-2.23409	-0.39062

RD-WO_2(nacnac)$_2$

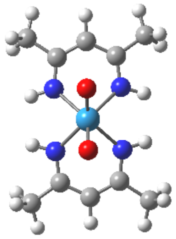

Atom	X	Y	Z
W	0.00002	0.00000	-0.58852
O	1.28600	0.00000	-1.73476
C	2.61139	-1.24386	0.60057
C	3.25186	0.00000	0.57731
H	4.32962	0.00000	0.68849
C	2.61139	1.24386	0.60057
C	-2.61153	-1.24383	0.60024
C	-3.25205	0.00000	0.57663
H	-4.32983	0.00000	0.68756
C	-2.61153	1.24383	0.60024
O	-1.28581	0.00000	-1.73491
H	-1.01744	2.35554	0.64134
N	-1.30275	1.40025	0.45330
N	1.30264	1.40033	0.45336
H	1.01738	2.35572	0.64099
C	-3.44300	-2.48102	0.84719
H	-3.00372	-3.08754	1.64750
H	-4.46971	-2.23776	1.12808
H	-3.46912	-3.09234	-0.06195
C	-3.44300	2.48102	0.84719
H	-3.46912	3.09234	-0.06195
H	-4.46971	2.23776	1.12808
H	-3.00372	3.08754	1.64750
C	3.44303	2.48105	0.84688
H	3.00186	3.09011	1.64420
H	4.46873	2.23775	1.13144
H	3.47234	3.08987	-0.06385
C	3.44303	-2.48105	0.84688
H	4.46873	-2.23775	1.13144
H	3.00186	-3.09011	1.64420
H	3.47234	-3.08987	-0.06385
N	-1.30275	-1.40025	0.45330
H	-1.01744	-2.35554	0.64134
N	1.30264	-1.40033	0.45336
H	1.01738	-2.35572	0.64099

B-WO_2(nacnac)$_2$

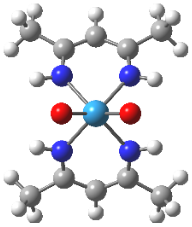

Atom	X	Y	Z
W	0.00000	0.00000	-0.96990
O	0.00000	-1.31072	-2.09900
C	2.46026	-1.21515	0.83300
C	2.93746	0.00000	1.32226
H	3.77811	0.00000	2.00443
C	2.46026	1.21515	0.83300
C	-2.46026	-1.21515	0.83300
C	-2.93746	0.00000	1.32226
H	-3.77811	0.00000	2.00443
C	-2.46026	1.21515	0.83300
O	0.00000	1.31072	-2.09900
N	1.43737	-1.29873	-0.00260
N	-1.43737	1.29873	-0.00260
H	1.29796	-2.24170	-0.35407
H	-1.29796	2.24170	-0.35407
C	3.15678	2.49814	1.21943
H	2.43419	3.22920	1.59723
H	3.91544	2.33182	1.98720
H	3.64647	2.93394	0.34113
C	3.15678	-2.49814	1.21943
H	2.43419	-3.22920	1.59723
H	3.64647	-2.93394	0.34113
H	3.91544	-2.33182	1.98720
C	-3.15678	-2.49814	1.21943
H	-3.91544	-2.33182	1.98720
H	-3.64647	-2.93394	0.34113
H	-2.43419	-3.22920	1.59723
C	-3.15678	2.49814	1.21943
H	-2.43419	3.22920	1.59723
H	-3.64647	2.93394	0.34113
H	-3.91544	2.33182	1.98720
N	1.43737	1.29873	-0.00260
H	1.29796	2.24170	-0.35407
N	-1.43737	-1.29873	-0.00260
H	-1.29796	-2.24170	-0.35407

trans-WO_2(nacnac)$_2$

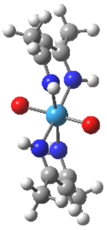

Atom	X	Y	Z
O	0.51360	-0.09112	1.68286
C	2.71015	1.16030	-0.01276
C	3.31480	-0.08543	0.32833
H	4.33945	-0.04558	0.68224
C	2.80727	-1.32047	-0.02107
C	-3.36861	0.00072	-0.34496
H	-4.39188	0.06341	-0.69954
C	-2.89475	-1.24256	0.02191
O	-0.56914	-0.10028	-1.69886
H	1.21402	2.14061	-0.82285
N	1.46055	1.25992	-0.38192
C	-2.73023	1.23429	-0.02138
C	3.69723	-2.53651	-0.02391
H	3.30201	-3.29058	0.66494
H	3.72616	-2.98419	-1.02493
H	4.72059	-2.29378	0.27001
C	3.60800	2.37364	-0.02417
H	3.95829	2.57446	0.99432
H	4.48928	2.20487	-0.65064
H	3.08375	3.26275	-0.38518
C	-3.81735	-2.43385	0.04179
H	-3.44273	-3.20815	-0.63605
H	-3.85840	-2.86622	1.04910
H	-4.83375	-2.16768	-0.25595
C	-3.59501	2.47146	-0.02661
H	-4.47853	2.33618	0.60484
H	-3.04593	3.35161	0.31910
H	-3.94303	2.66583	-1.04711
N	-1.62789	-1.43367	0.44986
H	-1.53193	-2.16882	1.14568
N	1.53568	-1.48317	-0.44652
H	1.41957	-2.22557	-1.13148
N	-1.47829	1.30514	0.34636
H	-1.20775	2.18499	0.77473
W	-0.02981	-0.25736	-0.00691

Δ-$WO_2(nacnac)_2^{Me4}$

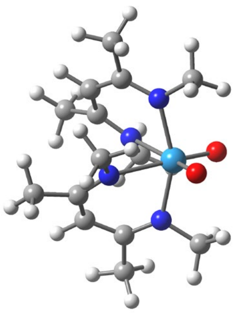

Atom	X	Y	Z
O	-0.26363	2.13567	-1.32498
C	2.52644	-0.57883	-0.72962
C	2.07794	-1.72157	-0.08092
H	2.58833	-2.64925	-0.31119
C	1.22713	-1.71875	1.05175
C	-1.22592	-1.71985	-1.05093
C	-2.07748	-1.72286	0.08108
H	-2.58797	-2.65062	0.31075
C	-2.52622	-0.58051	0.73038
O	0.26205	2.13645	1.32475
N	1.96552	0.63165	-0.59447
N	-1.96542	0.63012	0.59632
C	1.22189	-2.96092	1.91100
H	0.19495	-3.26638	2.13853
H	1.73039	-3.78670	1.40918
H	1.73162	-2.78194	2.86478
C	3.73643	-0.72387	-1.62527
H	3.59757	-0.19578	-2.57408
H	4.63505	-0.31763	-1.14728
H	3.92508	-1.77819	-1.83726
C	-1.21900	-2.96259	-1.90938
H	-1.72877	-3.78797	-1.40824
H	-1.72626	-2.78426	-2.86458
H	-0.19148	-3.26829	-2.13416
C	-3.73627	-0.72654	1.62582
H	-3.59769	-0.19912	2.57503
H	-4.63499	-0.32027	1.14805
H	-3.92452	-1.78108	1.83704
W	-0.00032	1.06415	0.00010
N	0.51213	-0.67213	1.38553
N	-0.51137	-0.67292	-1.38493
C	-2.67538	1.80819	1.10153
H	-2.41031	2.00720	2.14786
H	-2.38352	2.68073	0.51519
H	-3.75916	1.69825	1.01212
C	0.09448	-0.64660	-2.71075
H	0.93503	-1.34991	-2.78228
H	-0.63234	-0.89477	-3.49309
H	0.46345	0.35982	-2.90921
C	2.67516	1.81042	-1.09836
H	2.38320	2.68212	-0.51077
H	3.75897	1.70057	-1.00929
H	2.40974	2.01075	-2.14436
C	-0.09335	-0.64536	2.71147
H	-0.46172	0.36132	2.90987
H	-0.93430	-1.34819	2.78314
H	0.63342	-0.89402	3.49371

DML-$WO_2(nacnac)_2^{Me4}$

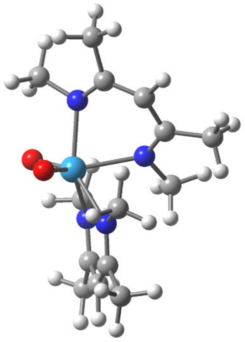

Atom	X	Y	Z
O	0.26984	-2.37320	-0.14050
O	-0.41238	-0.99444	1.99437
N	1.78488	-0.48249	-0.83891
N	-0.79054	0.96050	-0.94629
C	-1.92419	1.59207	-0.67566
C	-3.00657	0.96991	-0.03590
H	-3.87248	1.57805	0.19135
C	-3.13645	-0.41244	0.09012
C	2.11834	1.46162	1.35202
C	3.14583	0.91748	0.56549
H	4.15441	1.25827	0.76142
C	2.96363	0.03070	-0.49174
C	2.52055	2.53366	2.34271
H	2.15237	2.30419	3.34778
H	3.60683	2.63003	2.38654
H	2.10742	3.50841	2.05862
C	4.20270	-0.32227	-1.28913
H	4.28440	-1.40206	-1.44668
H	4.18954	0.15515	-2.27596

H	5.09918	0.01820	-0.76793
C	-4.51156	-0.94016	0.43062
H	-5.14963	-0.12534	0.77803
H	-4.99192	-1.38723	-0.44698
H	-4.46396	-1.70409	1.21219
C	-2.12498	3.04398	-1.05781
H	-1.25114	3.65013	-0.79997
H	-2.30644	3.15458	-2.13268
H	-2.99295	3.45007	-0.53480
W	-0.08522	-0.75750	0.32479
C	0.17590	1.64845	-1.80206
H	-0.30155	2.40224	-2.43111
H	0.96313	2.11951	-1.20398
H	0.65009	0.93018	-2.46889
C	1.74691	-1.29305	-2.05840
H	0.71976	-1.35981	-2.42607
H	2.34940	-0.84631	-2.85582
H	2.08539	-2.31866	-1.87713
N	-2.12565	-1.25593	-0.11063
N	0.85784	1.09179	1.24752
C	-2.42031	-2.69034	-0.13014
H	-3.40699	-2.89049	-0.55378
H	-1.66834	-3.19259	-0.73598
H	-2.36528	-3.10924	0.88252
C	-0.11283	1.76742	2.10527
H	-0.00510	2.85749	2.05675
H	-1.12847	1.52470	1.79117
H	-0.01889	1.44217	3.14792

CH-$WO_2(nacnac)_2^{Me4}$

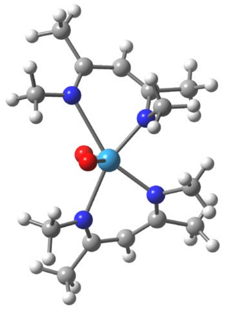

Atom	X	Y	Z
W	0.07905	-0.25834	0.29852
O	-0.00467	-0.77554	-1.34195
O	-0.13885	-1.30155	1.66952
C	-1.96818	3.07181	-0.78692
C	-2.10407	1.61382	-0.44102
C	-2.96018	0.79503	-1.07970
C	-3.19898	-0.61809	-0.74257
C	-4.52010	-1.20881	-1.18951
H	-2.11550	3.69992	0.09976
H	-0.95221	3.26295	-1.15415
H	-2.68237	3.38313	-1.55416
H	-3.57203	1.21115	-1.87539
H	-5.05356	-0.53536	-1.86433
H	-4.36525	-2.16434	-1.70167
H	-5.16775	-1.40164	-0.32564
C	-1.80047	1.48061	1.94176
H	-1.74785	2.55875	2.15244
H	-1.22460	0.95293	2.70543
H	-2.84707	1.15805	2.03377
C	-2.56257	-2.65438	0.30315
H	-2.35688	-3.32398	-0.54274
H	-3.58883	-2.83912	0.64738
H	-1.86424	-2.89371	1.10821
N	-2.32332	-1.28329	-0.08819
N	-1.29024	1.14813	0.61728
C	4.15926	-1.87217	-1.01759
C	3.03483	-0.94691	-0.61233
C	3.09895	0.39264	-1.09144
C	2.46597	1.44568	-0.47142
C	2.92453	2.84837	-0.79989
H	4.85863	-2.02215	-0.18659
H	3.77730	-2.85503	-1.31026
H	4.72102	-1.45243	-1.85473
H	3.84147	0.62164	-1.84615
H	3.49088	2.84785	-1.73359
H	2.07621	3.53218	-0.90706
H	3.57856	3.25047	-0.01765
C	2.18337	-2.65752	0.80968
H	1.82913	-3.42936	0.11270
H	1.53412	-2.67056	1.68446
H	3.20605	-2.90362	1.11493
C	1.22637	2.37893	1.37545
H	0.52485	3.11695	0.96617
H	2.15646	2.88114	1.65678
H	0.79122	1.98161	2.29459
N	1.47897	1.28339	0.44520
N	2.08973	-1.34028	0.19576

RD-$WO_2(nacnac)_2^{Me4}$

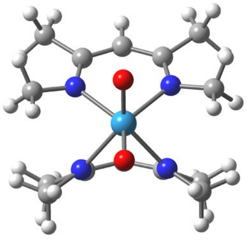

Atom	X	Y	Z
W	-0.22321	1.16607	0.16947
O	-1.49548	1.95506	1.03289
O	0.54272	2.62973	-0.33136
C	3.12410	-1.59882	0.78156
C	2.00013	-0.81676	0.14772
C	1.17315	-1.47013	-0.79495
C	0.61836	-0.79698	-1.90790
C	0.45060	-1.56032	-3.19857
H	4.08886	-1.29072	0.36082
H	3.16821	-1.41969	1.86172
H	3.00773	-2.67024	0.60498
H	1.24996	-2.54985	-0.85696
H	0.55986	-2.63505	-3.03821
H	-0.53032	-1.36605	-3.64662
H	1.20875	-1.24959	-3.92753
C	-0.92003	-1.19922	2.14453
C	-2.33594	-1.17914	0.03852
C	-0.78443	-1.94225	3.45791
C	-1.84325	-1.73252	1.23110
C	-3.50700	-1.90421	-0.59266
H	-1.20312	-1.35221	4.28130
H	-1.31617	-2.89442	3.42441
H	0.26466	-2.14235	3.69959
H	-2.32374	-2.65054	1.54539
H	-3.68382	-2.86211	-0.10108
H	-4.42290	-1.30822	-0.50696
H	-3.33674	-2.09057	-1.65817
C	-2.60110	0.45343	-1.68957
H	-2.51132	-0.17155	-2.58932
H	-2.21582	1.44506	-1.93380
H	-3.66415	0.57997	-1.45857
C	2.94878	1.24969	0.94296
H	3.19732	0.96524	1.97498
H	2.65488	2.30081	0.92271
H	3.85315	1.13737	0.33275
C	0.25769	1.28817	-3.05874
H	-0.60587	1.01879	-3.68276
H	1.16367	1.17696	-3.66675
H	0.17351	2.33613	-2.76496
C	0.59499	0.40831	3.06281
H	1.45492	-0.22887	3.31319
H	-0.01966	0.53201	3.96080
H	0.97783	1.39861	2.80951
N	0.33942	0.49242	-1.84814
N	1.85146	0.47104	0.39988
N	-1.86685	-0.09630	-0.55735
N	-0.18114	-0.12057	1.94873

B-$WO_2(nacnac)_2^{Me4}$

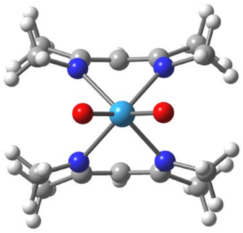

Atom	X	Y	Z
W	0.00299	1.23726	-0.00145
O	-0.69656	2.41304	-1.04881
O	0.70508	2.41341	1.04379
C	3.19507	-1.81269	0.79423
C	2.15693	-1.02208	0.03123
C	1.46223	-1.69350	-0.97799
C	0.79176	-1.02343	-2.00444
C	0.48064	-1.81553	-3.25374
H	4.20805	-1.53851	0.47856
H	3.12729	-1.62724	1.87103
H	3.06728	-2.88207	0.61430
H	1.62378	-2.75844	-1.08566
H	0.59882	-2.88466	-3.06586
H	-0.54116	-1.63086	-3.60053
H	1.15760	-1.54226	-4.07106
C	-0.79043	-1.01826	2.00551
C	-2.15564	-1.01741	-0.03013
C	-0.48100	-1.80893	3.25613
C	-1.46225	-1.68863	0.98012
C	-3.19540	-1.80713	-0.79185
H	-1.15731	-1.53277	4.07301
H	-0.60155	-2.87812	3.07008
H	0.54122	-1.62592	3.60256
H	-1.62594	-2.75308	1.08948

H	-3.06956	-2.87650	-0.61047
H	-4.20781	-1.53065	-0.47634
H	-3.12750	-1.62329	-1.86891
C	-2.96038	0.92685	-1.14579
H	-2.85741	0.64195	-2.20075
H	-2.81388	2.00270	-1.08492
H	-3.97645	0.68124	-0.81844
C	2.96549	0.92223	1.14400
H	2.86206	0.63877	2.19931
H	2.82086	1.99826	1.08184
H	3.98110	0.67438	0.81691
C	0.06713	0.91948	-3.17623
H	-0.94900	0.63616	-3.47938
H	0.75411	0.67051	-3.99227
H	0.07038	1.99572	-3.02001
C	-0.06184	0.92513	3.17403
H	0.95362	0.64008	3.47782
H	-0.74948	0.67918	3.99042
H	-0.06266	2.00110	3.01592
N	0.46679	0.26445	-1.93234
N	1.96624	0.26600	0.30322
N	-1.96236	0.26986	-0.30415
N	-0.46281	0.26880	1.93126

Cis-Δ-$WO_2(nacnac)_2^{Ph4}$

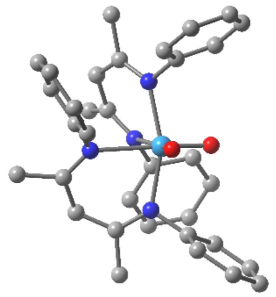

Atom	X	Y	Z
O	-0.19800	-2.01738	-1.32711
C	-2.66236	0.63668	0.49817
C	-2.03047	1.76689	0.99799
H	-2.65591	2.64085	1.13166
C	-0.76970	1.79665	1.63719
C	0.77024	1.79377	-1.63933
C	2.03094	1.76459	-1.00000
H	2.65668	2.63814	-1.13496
C	2.66241	0.63499	-0.49830
O	0.19716	-2.01519	1.33136
N	-2.04467	-0.52118	0.21320
N	2.04441	-0.52230	-0.21168
C	-0.52392	2.96361	2.57071
H	0.36603	3.52538	2.27359
H	-1.37836	3.64254	2.57935
H	-0.35111	2.60702	3.59107
C	-4.15769	0.71715	0.29697
H	-4.42363	0.48164	-0.73744
H	-4.67164	-0.00735	0.93641
H	-4.52347	1.71651	0.53944
C	0.52481	2.95937	-2.57463
H	1.37961	3.63783	-2.58462
H	0.35141	2.60133	-3.59438
H	-0.36473	3.52204	-2.27800
C	4.15775	0.71533	-0.29701
H	4.42342	0.48168	0.73789
H	4.67157	-0.01053	-0.93499
H	4.52393	1.71411	-0.54127
W	-0.00020	-0.94913	0.00121
N	0.10998	0.82563	1.51804
N	-0.10976	0.82323	-1.51866
C	1.16920	0.77107	2.47924
C	2.28030	1.61305	2.41045
C	1.08744	-0.16610	3.51162
C	3.29945	1.51154	3.35323
H	2.34664	2.33496	1.60372
C	2.10522	-0.26093	4.45555
H	0.23718	-0.83711	3.54015
C	3.21837	0.57323	4.37888
H	4.16055	2.17019	3.28254
H	2.03028	-0.99900	5.24864
H	4.01551	0.49283	5.11176
C	2.86684	-1.68357	0.01737
C	3.38886	-1.94393	1.28312
C	3.11607	-2.55522	-1.03916
C	4.18384	-3.06668	1.47949
H	3.15879	-1.27267	2.10353
C	3.91066	-3.67992	-0.83518
H	2.66572	-2.35294	-2.00590
C	4.44941	-3.93549	0.42248
H	4.59089	-3.26737	2.46610
H	4.10124	-4.36007	-1.65995
H	5.06618	-4.81491	0.58226
C	-1.16899	0.76743	-2.47977

C	-2.27978	1.60995	-2.41243
C	-1.08759	-0.17156	-3.51052
C	-3.29896	1.50719	-3.35503
H	-2.34582	2.33329	-1.60696
C	-2.10540	-0.26764	-4.45428
H	-0.23758	-0.84295	-3.53787
C	-3.21823	0.56708	-4.37906
H	-4.15981	2.16630	-3.28550
H	-2.03075	-1.00712	-5.24608
H	-4.01541	0.48570	-5.11180
C	-2.86748	-1.68260	-0.01389
C	-3.11715	-2.55231	1.04412
C	-3.38942	-1.94499	-1.27926
C	-3.91211	-3.67708	0.84200
H	-2.66683	-2.34852	2.01056
C	-4.18477	-3.06780	-1.47377
H	-3.15900	-1.27524	-2.10081
C	-4.45080	-3.93466	-0.41528
H	-4.10305	-4.35572	1.66794
H	-4.59175	-3.27007	-2.46009
H	-5.06785	-4.81413	-0.57360

DML-$WO_2(nacnac)_2^{Ph4}$

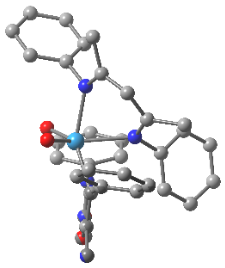

Atom	X	Y	Z
O	0.13783	-2.23797	-0.62485
O	0.59358	-0.20951	-2.19596
N	-1.79537	-1.00841	0.49865
N	0.55676	0.96797	1.06216
C	1.56856	1.82131	0.91914
C	2.67727	1.54557	0.10360
H	3.40018	2.33662	-0.04811
C	3.06650	0.23738	-0.16600
C	-2.54616	1.31014	-1.24132
C	-3.40886	0.37660	-0.65430
H	-4.46727	0.51742	-0.83075
C	-3.05060	-0.65828	0.21067
C	-3.19951	2.43964	-2.01191
H	-2.69980	2.59889	-2.97145
H	-4.25362	2.21982	-2.19219
H	-3.13348	3.38178	-1.45830
C	-4.19960	-1.38655	0.87799
H	-4.18132	-2.45659	0.65375
H	-4.12797	-1.29042	1.96608
H	-5.15647	-0.97532	0.55179
C	4.51414	-0.03119	-0.49240
H	5.05845	0.90734	-0.61262
H	4.98699	-0.61133	0.30641
H	4.60255	-0.61924	-1.41058
C	1.63518	3.10498	1.71680
H	0.64867	3.46559	2.01124
H	2.22444	2.95713	2.62797
H	2.12912	3.87119	1.11424
W	0.15588	-0.53120	-0.57150
N	2.20869	-0.78510	-0.07733
N	-1.22879	1.26728	-1.11006
C	2.74563	-2.11268	-0.02101
C	3.05186	-2.66526	1.21870
C	2.95210	-2.84622	-1.18813
C	3.58226	-3.95063	1.29274
H	2.85638	-2.08671	2.11678
C	3.48729	-4.12618	-1.10945
H	2.66948	-2.40959	-2.14106
C	3.80601	-4.68068	0.12933
H	3.81479	-4.38213	2.26182
H	3.64679	-4.69785	-2.01890
H	4.21858	-5.68355	0.18663
C	-0.23202	1.12615	2.24165
C	0.36124	0.85823	3.47795
C	-1.56253	1.54231	2.19735
C	-0.36040	1.01341	4.65671
H	1.38937	0.50836	3.50002
C	-2.28367	1.69132	3.37813
H	-2.03582	1.72032	1.24094
C	-1.68860	1.43092	4.61071
H	0.11235	0.79678	5.61005
H	-3.32109	2.00889	3.33000
H	-2.25771	1.54632	5.52805
C	-0.51212	2.42735	-1.54879
C	-0.47106	3.54569	-0.71212
C	0.13549	2.48491	-2.78520
C	0.22144	4.69219	-1.08688
H	-0.98776	3.50672	0.24216
C	0.83132	3.63153	-3.15559

H	0.10135	1.62247	-3.43725
C	0.88470	4.73651	-2.30958
H	0.24239	5.54934	-0.41941
H	1.33405	3.65973	-4.11805
H	1.43067	5.62749	-2.60541
C	-1.65870	-2.12974	1.39522
C	-1.72226	-3.43796	0.90285
C	-1.48956	-1.92909	2.76262
C	-1.59126	-4.51918	1.76307
H	-1.85895	-3.59264	-0.16105
C	-1.35462	-3.01713	3.62412
H	-1.49112	-0.92467	3.16087
C	-1.39804	-4.31433	3.12913
H	-1.63403	-5.52821	1.36358
H	-1.22315	-2.83867	4.68770
H	-1.29079	-5.16210	3.79953

CH-WO$_2$(nacnac)$_2^{Ph4}$

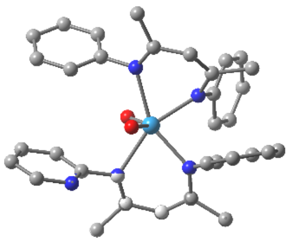

Atom	X	Y	Z
W	0.09160	-0.15537	-0.02409
O	0.92870	0.37739	1.38845
O	0.86987	-0.55662	-1.51877
C	0.93331	3.56678	-2.53227
C	0.22415	2.48172	-1.75824
C	-1.14860	2.23164	-2.07133
C	-2.03013	1.75412	-1.13706
C	-3.50748	1.99379	-1.36855
H	1.05921	4.45798	-1.90847
H	1.93269	3.23890	-2.83150
H	0.36322	3.84500	-3.42111
H	-1.55124	2.67531	-2.97473
H	-3.79440	1.49860	-2.30181
H	-4.12264	1.58772	-0.56640
H	-3.72456	3.06134	-1.47079
C	-1.59219	-2.62711	1.02482
C	0.81183	-2.89453	1.50846
C	-2.93224	-3.30740	1.19707
C	-0.56149	-3.07319	1.82464
C	1.80172	-3.83225	2.16079
H	-3.28845	-3.72251	0.24997
H	-2.84806	-4.12051	1.91992
H	-3.69451	-2.60781	1.55290
H	-0.79805	-3.80558	2.58554
H	1.30802	-4.47533	2.89174
H	2.28387	-4.46241	1.40665
H	2.59518	-3.26820	2.65950
N	-1.44045	-1.65103	0.09599
N	-1.60856	1.09664	-0.00945
N	1.20588	-2.02095	0.62404
N	0.81272	1.85751	-0.78103
C	-2.22096	1.44219	1.22219
C	-2.62360	2.76886	1.43834
C	-2.38118	0.52442	2.26777
C	-3.21871	3.15063	2.63351
H	-2.44328	3.50812	0.66352
C	-2.96122	0.91817	3.46927
H	-2.04270	-0.49662	2.15758
C	-3.39727	2.22533	3.65854
H	-3.52527	4.18378	2.77011
H	-3.06993	0.18694	4.26504
H	-3.85334	2.52397	4.59726
C	2.57587	-2.01456	0.22003
C	2.96091	-2.72337	-0.91743
C	3.51632	-1.27742	0.93781
C	4.29036	-2.71026	-1.32394
H	2.20629	-3.26211	-1.48098
C	4.84691	-1.27527	0.52941
H	3.18532	-0.68682	1.78520
C	5.23873	-1.99029	-0.59874
H	4.58622	-3.26067	-2.21237
H	5.57466	-0.69586	1.08983
H	6.27722	-1.98294	-0.91701
C	2.11113	2.26392	-0.35724
C	3.23773	1.61481	-0.85997
C	2.24526	3.28442	0.58200
C	4.50220	2.01389	-0.43887
H	3.10581	0.78809	-1.55137
C	3.51278	3.67271	1.00233
H	1.35139	3.74918	0.98683
C	4.64473	3.04317	0.48814
H	5.37749	1.50355	-0.82961
H	3.61621	4.46530	1.73774
H	5.63386	3.34868	0.81667
C	-2.40286	-1.56977	-0.96471

C	-3.72205	-1.16746	-0.74665
C	-1.99177	-1.89479	-2.25855
C	-4.63144	-1.15009	-1.79783
H	-4.02526	-0.84992	0.24580
C	-2.89871	-1.84963	-3.31353
H	-0.95394	-2.16474	-2.42464
C	-4.22446	-1.49152	-3.08570
H	-5.65709	-0.84397	-1.61258
H	-2.56616	-2.10359	-4.31558
H	-4.93452	-1.46868	-3.90700
